# 

**Published:** 1911-05-06

**Authors:** 


### BOOKS RECEIVED.

Constable and Co., Ltd.

"Clinical Lectures on the Acute Abdomen." W. H. Battle, F.R.C.S.

Eyre and Spottiswoode.

"Our Bible and the Ancient Manuscripts." F. J. Kenyon, M.A.

The opening ceremony of the Cancer Hospital Research Institute has been postponed owing to the indisposition of H.R.H. the Duke of Connaught. The altered date will be announced in due course.

---

The new children's ward of Beth Israel Hospital, New York, which has been built on the roof of the main wing of the hospital building, accommodates twenty-five patients and comprises an open-air and an enclosed part, together with a special-diet kitchen, an isolation ward with four beds, and outdoor and indoor playgrounds. The cost of constructing the ward was about $10,000.

### BUILDINGS CONTEMPLATED AND IN PROGRESS.

Falkirk.—Messrs. A. and W. Black are architects for the new block to be erected at Camelon Hospital. Quantities for tendering may be obtained from them.

Goole.—Additions and alterations are proposed to the workhouse hospital. Quantities may be obtained from the architects, Messrs. Thorpe and Turner, Carlisle Street, Goole.

Hinckley.—Mr. W. T. Grewcock, A.R.I.B.A., is architect for the additions to the workhouse at Hinckley. The lowest tender of sixteen is that by F. Sleath, amounting to £5,965; the highest is £7,200.

Huntingdon.—Tenders are invited for additions to the hospital. Mr. S. Inskip Ladds, A.R.I.B.A., of Huntingdon, is the architect.

Lewisham.—At a meeting of the Joint Committee of St. John's Hospital and the King Edward Memorial Fund it was decided to erect as a memorial a bacteriological and *x*-ray department at St. John's Hospital.

Norfolk.—As a King Edward memorial it has been decided to remodel the West Norfolk and Lynn Hospital. Messrs. Macalister and Skipper are the architects.

Southampton.—A new out-patients' department is projected for the Royal South Hants and Southampton Hospital. Tenders are to be in before May 8.

Wimbledon.—A new hospital is projected at Merton, and the foundation-stone will be laid next month. Of the £10,000 required, £7,000 has been collected. The Council of the King's Fund has promised £1,000.

### JOTTINGS.

A strong branch of the Hospital Saturday Fund will shortly be established in the City.

The Queen has sent a large hamper of primroses for distribution among the patients of the Victoria Hospital for Children at Chelsea.

The Kilburn Provident Dispensary, which has over 3,000 cases a year and dispenses over 13,000 prescriptions, celebrates its jubilee this week.

Princess Henry of Battenburg will attend a matinée at the Court Theatre on Wednesday, May 10, in aid of the Queen's Hospital for Children, Hackney Road.

Mr. John C. Nicholson has given £1,000 towards the £150,000 King Edward Memorial Fund at Leeds, and Mrs. A. S. Macpherson £500. Practically £100,000 has been raised.

The Costume Skating Fête which Prince Alexander of Teck's influential committee organised last Monday in aid of the Prince Francis of Teck Memorial Fund was a great success. The Royal party, who arrived at 9.30 p.m., were received by the Mayor of Paddington. Supper was served soon after 11 p.m. Among the most successful costumes were a thistle, a Rajah, a Mephistopheles, and a lady disguised as a chimney sweep. "Votes for Women" and an "enthusiastic beginner" bandaged from head to foot created much laughter.

The Canadian Memorial to King Edward, according to the Toronto correspondent of the *Times*, is taking the form of an organisation to raise a million dollars (£200,000) for a national sanatorium for consumptives.

The Duke and Duchess of Sutherland will lend Stafford House for an afternoon concert on May 9, which has been arranged by Miss Nora Johnston in aid of the Prince Francis of Teck Memorial Fund.

The annual meeting of the Medical Mission Hospital, Plaistow, of the Canning Town Women's Settlement, will be held at Toplady Hall, Whitefield's, Tottenham Court Road, on Thursday, May 11, 1911, at 3 p.m.

As an instance of what a little enterprise can do, it is interesting to note that the Leicester Amateur Musical and Dramatic Society, in addition to other contributions to charitable effort, is able to maintain a Cot in the Children's Hospital attached to the Leicester Infirmary.

Owing to the coolness and bravery of the hospital staff and attendants of the Philadelphia Hospital, the fire which broke out in the institution on April 6 was unattended by loss of life. Two hundred patients in the tuberculosis pavilion were carried out through dense smoke. The fire broke out in the laundry, and for a time threatened the whole building. The damage done is estimated at 285,000 dollars, or roughly, £57,000.

### Gifts and Bequests.

Mr. Marcus Nathan Adler, of Craven Hill, W., former Vice-President of the Institute of Actuaries, has left £100 each to the Jewish Convalescent Homes at Brighton and Daneswood, and the London Hospital.

Mr. W. Bridgeman, M.P., Hon. Treasurer of the Florence Nightingale Hospital, Lisson Grove, has received a second anonymous £500 for the building fund.

The Queen's Fund for the Maintenance of Queen Victoria's Jubilee Institute for Nurses has received an anonymous donation of £1,000.

The Treasurer of the Bradford Royal Infirmary has received £1,000 from Mrs. John Lister Margerison to endow a bed as a perpetual memorial to her late husband, who was for many years a subscriber to the institution.

Mr. Jonas Hill, of Summerbank, Gravesend, has bequeathed £300 to the Gravesend Hospital.

Mr. Charles Thomas Robinson, of King's Lynn, Norfolk, wine and spirit merchant, has left £100 to the Lynn Hospital and £100 to the Hunstanton Convalescent Home.

The Clothworkers' Company has sent £100 to St. Mary's Hospital, Paddington.

Mr. George Harding, of Stanton, Bebington, shipowner, who left estate sworn at £433,768 gross, has left £5,000 for charitable purposes, free of duty, the bequests including £1,000 each to the Royal Infirmary, Liverpool, the Royal Southern Hospital, Liverpool, and the David Lewis Hospital, Liverpool; and £500 to the Stanley Hospital, Liverpool.

Mrs. M. A. Hodgson, of Leicester, has left £50 to the Leicester Infirmary, which also has received £50 from the Ellistown Colliery Workmen's Accident Fund.

Mr. William Young, of Salisbury, has left £1,500 to the Cancer Hospital, Brompton; £500 to the St. Bartholomew's Hospital, and £250 each to the Southampton Free Eye Hospital and the Royal Hospital for Incurables.

Mr. William Woolland, draper, of Knightsbridge, has bequeathed £1,000 to King Edward's Hospital Fund for London, £500 each to the Royal Devon Hospital, Exeter, and to the Royal Hospital, Winchester.

Miss Kate Anne Walker has bequeathed £300 to the Stockton-on-Tees Hospital.

Miss Margaret Barwise, of Carlisle, has bequeathed £100 each to the Cumberland Infirmary, Carlisle, the Border Counties Home for Incurables, Carlisle, and the Cumberland and Westmorland Convalescent Institution at Silloth.

The Goldsmiths' Company and Mr. W. Vivian have sent £150 and £50 respectively to the Royal Sea Bathing Hospital for Surgical Tuberculosis, Margate.

Miss Sarah Hampson, of Radcliff, Lancs, who left estate valued at £331,036 gross, has bequeathed £1,000 each to the Manchester Royal Infirmary, the Bury Infirmary, the Southport Infirmary, the Pendlebury Children's Hospital, the Radcliffe and Whitefield Consumptives' Relief Fund.

The Salford Royal Hospital and the Teddy Beswick Benefit Fund has received £50 as part proceeds of the Salford and Swinton Veterans' football match.

The Bradford Royal Infirmary has received £318 from Mr. Herbert J. Jeffrey, in payment of half of the residue of the estate of the late Mrs. Elizabeth Wright of Bradford. The residue was left for division between the Bradford Royal Infirmary and the Bradford Institution for the Blind in such shares as her trustees might think fit.

King Edward's Hospital Fund for London has received £500 from the Clothworkers' Company.

Mrs. Sarah Martha Packe, of Hyde Park, who left estate valued at £111,331 gross, has bequeathed £500 each to the Royal Free Hospital and to the London Hospital.

Mr. Charles Sexton, of Dublin, has left £1,000 each to the City of Dublin Royal Hospital, the Meath Hospital, the Richmond Hospital, Sir Patrick Dun's Hospital, the Cork Street Fever Hospital, and the Mercers' Hospital; and £500 each to the Adelaide Hospital, the Jervis Street Hospital, the National Institution and Molyneux Asylum for the Female Blind, the Richmond National Institution for the Blind, the National Children's Hospital, the Hospital for Incurables, all of Dublin.

The will of the late Miss Isabel Goldsmid, of London, which is about to be proved, contains the following charitable legacies: £500 each to the University College Hospital, London, the Brompton Consumption Hospital, the North-Eastern Hospital for Children, Hackney Road, N.E.; £300 each to the North London Hospital for Consumption and Diseases of the Chest, Mount Vernon, Hampstead Heath, and the same hospital, Northwood, Middlesex; and £200 each to the Ladies' Association of the Metropolitan Hospital, Kingsland Road, N., and the Jewish Home for Consumptives, Daneswood, Bedfordshire.

Lord Donoughmore, Treasurer of the London Homœopathic Hospital, Great Ormond Street, W.C., has received £100 from Mr. J. Oxley Laurie towards the appeal for £8,000 to complete the building of a new Home for seventy-five nurses of the hospital. King Edward's Hospital Fund have also made a grant of £500 for the purpose.

Among the latest contributions received at the Bank of England for King Edward's Hospital Fund for London are: Annual subscriptions—Messrs. Speyer Brothers, £250; the Duke of Norfolk, £50; Lord Wemyss and March, £50. Donations—"A.," £1,000; Messrs. Crosse and Blackwell, Limited, £105; "E. H. B.," £100.

The first replies to the appeal for funds to clear off the debt of the North Ormesby Hospital, Middlesbrough, include Mr. Alfred O. Cochrane, treasurer, £100; Cochrane and Co., Ltd., Middlesbrough, £150; Connal and Co., Ltd., Middlesbrough, £5 5s.; Dorman, Long and Co., Ltd., Middlesbrough, £450 (spread over three years); and Redcar, Coatham, Marske and Saltburn Gas Company, Ltd., £10 10s.

The Rev. Henry Drayton Wyatt, of Aldershot, has bequeathed a freehold ground-rent at Woolwich producing £30 per annum to his niece for life, with remainder to the Royal Surrey County Hospital, Guildford, and to his cook a life interest in a trust fund of £500, with remainder to the same hospital; to the Aldershot Cottage Hospital the proceeds of the sale of his residence Drayton Lodge; £300 each to the Great Northern Hospital, London, and the Brompton Cancer Hospital; £200 each to the Royal Hospital for Incurables, Putney, the Royal Asylum for the Deaf and Dumb, the Aldershot Cottage Hospital, the Royal Surrey County Hospital, Guildford; and £100 to the vicar and churchwardens of Wyke, Surrey, to apply the income in keeping in order the grave of his wife, and, subject thereto, for the benefit of deserving widows of the parish, but should the vicar and churchwardens fail to keep the grave in order this bequest is to revert to the Aldershot Cottage Hospital. The residue of his property he left equally between the Great Northern Hospital, London, and two other charities. He expressed the desire that the benefits to the Aldershot Cottage Hospital should, as far as possible, be devoted to the endowment of a bed in memory of his late wife and himself.

### ANSWERS TO CORRESPONDENTS.

F. Noding.—All the large London hospitals possess installations for electric treatment. The electrical department at the London Hospital and at Charing Cross Hospital are excellent for the purpose you mention. We cannot recommend private institutions

Inquirer.—The book referred to was Albert Grotjahn's manual on hospitals and the insurance system ("Krankenhauswesen und Heilstattenbewegung im Lichte der sozialen Hygiene"). A copy of it is not in the British Museum Library, but may be seen at our Office.

### COMING EVENTS OF THE WEEK.

[Communications for this column must be sent to 29 Southampton Street, Strand, before noon on Wednesday.]

Saturday, May 6.—Constance, Countess of Derby opens the new buildings at the Bromley Cottage Hospital, and receives purses towards the completion of the Enlargement Fund.

French Hospital, Anniversary Dinner, French Ambassador presides, Hotel Cecil, 7.30 p.m.

Tuesday, May 9.—Opening of new extension to out-patient department and the open-air shelter by Princess Marie Louise of Schleswig Holstein, 3.15 p.m. for 3.30 p.m.

British Medical Temperance Association : Annual meeting, London Temperance Hospital, 4.30 p.m.

Thursday, May 11.—Annual Meeting of the British Home and Hospital for Incurables, at the Institution, Crown Lane, Streatham, 3 p.m.

Friday, May 12.—The Duchess of Connaught presents the St. John Ambulance Association Challenge Shields and Prizes, Portman Rooms.

Special matinée in aid of the Miller General Hospital for South-East London, The Playhouse, 2.30 p.m.

German Hospital, Dalston : Anniversary Dinner, Sir F. C. Lascelles presides, Whitehall Rooms, Hotel Metropole, 7 p.m.

### APPOINTMENTS VACANT.

Full particulars of these vacancies can be obtained on reference to our advertisement columns :

Beckett Hospital, Barnsley.—Second House Surgeon.

Leeds General Infirmary (Ida and Robert Arthington Hospital.—Medical Officer, Obstetric Officer, Two House Physicians, One House Surgeon, One Ophthalmic House Surgeon.

Royal Albert Hospital, Devonport.—Assistant Resident Medical Officers.

### THE QUESTION BOX.

### CHRISTENING A WARD.

Hospital Secretary writes : I shall be glad if you will kindly inform me if you know of any set "christening" ceremony in connection with the naming of a ward.

[Will any secretary who has had such a ceremony performed kindly send us a copy?—Ed. The Hospital.]

### THE WEEK'S NOVELTIES.

Spring is with us, and with it, in home and institution, naturally spring-cleaning. If one could offer a magic wand that would do all the cleaning, one could anticipate a large demand for it. Failing this, we must use the best of those aids that will accomplish the most with the least trouble. Of these, GLOBE METAL POLISH is the one that will be of the greatest assistance in the polishing of brasses and bright metals. It has exceptional properties in that it readily removes tarnish without the necessity of a lot of hard rubbing, and not only does it give a most brilliant shine, but the shine withstands the action of the atmosphere to a greater extent than that produced by any other means, so that the metals retain their brilliancy much longer. Globe is obtainable in both paste and liquid. For répousse or ornamental metals Globe liquid will be found a distinct convenience.

